# Human adipose tissue-derived stem cell extracellular vesicles attenuate ocular hypertension-induced retinal ganglion cell damage by inhibiting microglia- TLR4/MAPK/NF-κB proinflammatory cascade signaling

**DOI:** 10.1186/s40478-024-01753-8

**Published:** 2024-03-19

**Authors:** Shangli Ji, Yanfang Peng, Jian Liu, Pang Xu, Shibo Tang

**Affiliations:** 1 Aier Eye Hospital, Jinan University, 510632 Guangzhou, Guangdong China; 2 Aier Eye Institute, 410015 Changsha, Hunan China; 3 Department of Ophthalmology, The Second Xiangya Hospital, Central South University, 410011 Changsha, Hunan China; 4 Guangzhou Aier Eye Hospital, 510010 Guangzhou, Guangdong China

**Keywords:** Adipose tissue-derived extracellular vesicles, Microglia, Neuroinflammation, Ocular hypertension, TLR4/p-38 MAPK/NF-κB proinflammatory cascade response

## Abstract

Microglia-mediated neuroinflammatory responses are recognized as a predominant factor during high intraocular pressure (IOP)-induced retinal and optic nerve injury along with potential therapeutic targets for the disease. Our previous research indicated that mesenchymal stem cell (MSC) treatment could reduce high IOP-induced neuroinflammatory responses through the TLR4 pathway in a rat model without apparent cell replacement and differentiation, suggesting that the anti-neuroinflammatory properties of MSCs are potentially mediated by paracrine signaling. This study aimed to evaluate the anti-neuroinflammatory effect of human adipose tissue-derived extracellular vesicles (ADSC-EVs) in microbead-induced ocular hypertension (OHT) animals and to explore the underlying mechanism since extracellular vesicles (EVs) are the primary transporters for cell secretory action. The anti-neuroinflammatory effect of ADSC-EVs on LPS-stimulated BV-2 cells in vitro and OHT-induced retinal and optic nerve injury in vivo was investigated. According to the in vitro research, ADSC-EV treatment reduced LPS-induced microglial activation and the TLR4/NF-κB proinflammatory cascade response axis in BV-2 cells, such as CD68, iNOS, TNF-α, IL-6, and IL-1β, TLR4, p-38 MAPK, NF-κB. According to the in vivo data, intravitreal injection of ADSC-EVs promoted RGC survival and function, reduced microglial activation, microglial-derived neuroinflammatory responses, and TLR4/MAPK/NF-κB proinflammatory cascade response axis in the OHT mice. Our findings provide preliminary evidence for the RGC protective and microglia-associated neuroinflammatory reduction effects of ADSC-EVs by inhibiting the TLR4/MAPK/NF-κB proinflammatory cascade response in OHT mice, indicating the therapeutic potential ADSC-EVs or adjunctive therapy for glaucoma.

**Supplementary Information:**

The online version contains supplementary material available at 10.1186/s40478-024-01753-8.

## Introduction

Glaucoma, a progressive neurodegenerative disease, is among the most common causes of irreversible blindness [1]. Glaucoma is characterized by the progressive loss of retinal ganglion cells (RGCs; soma), thinning of the retinal nerve fiber layer (RNFL; RGC axons) and the inner plexiform layer (IPL; RGC dendrites) [2]. Glaucoma is a multifactorial disease, with old age, high intraocular pressure (IOP), and genetic predisposition as the main risk factors [3]. The underlying mechanisms, nevertheless, are largely unknown.

Accumulating evidence has demonstrated that neuroinflammation (inflammatory response in the nervous system) is involved in the pathological process of glaucoma. Neuroinflammatory responses mediated by microglia may be crucial in restoring retina homeostasis and defending against different retinal injuries [4]. Microglia are resident immune retinal macrophage-like cells involved in both innate and adaptive immunity. Under physiological conditions, they display a ramified quiescent phenotype with phagocytosis and clearance of cellular debris [5]. Ameboid-activated microglia and elevated expression of proinflammatory cytokines in postmortem glaucoma eyes have been reported [2, 6]. Activated and proliferative microglia have been noted in the optic nerve and retina of experimental glaucoma animal models in the early stages of the disease, before RGC and axon loss [7, 8]. Activated microglia can help clear cellular debris and secrete neuroprotective factors; however, with chronic persistent activation, their activation become irreversible and trigger secondary retinal and optic nerve damage. By modifying microglial activation, depleting microglia, and blocking microglial downstream, targeting microglia delays neurodegenerative processes in glaucoma animals [7–10]. Taken together, previous studies suggest the potential treatment strategies to target microglia in glaucoma. Mesenchymal stem cells (MSCs) show promising tissue protective effects in different eye disorders through immunosuppression, anti-inflammation, and trophic factor secretion [11, 12]. We have previously reported the neuroprotective and anti-neuroinflammatory effects of MSCs in a microsphere-induced OHT model in rodents [13]. As most of these protective effects of MSCs have been widely reported through paracrine actions rather than direct replacement or cell differentiation [14, 15], MSC-derived extracellular vesicles (MSC-EVs) have increasingly gained attention over the past decades. EVs, a range of membrane-bound biomaterials of different sizes and contents, can be released from all eukaryotic cells for intercellular communication and targeting cell modulation [16]. MSC-EVs carry information from the parental MSCs, such as cytosolic proteins, growth factors, lipids, and genetic factors, overcoming the defects of limited survivability, immunogenicity, and uncertainty of MSC fate after transplantation [17]. Recent work has demonstrated that MSC-EVs can repair ocular damage and restore vision, such as in corneal diseases, autoimmune uveitis, retinal diseases, glaucoma, idiopathic macular hole, and optic nerve crush [18, 19]. The underlying mechanisms, nevertheless, remain largely unknown. We previously demonstrated that intravitreally injected MSCs could reduce OHT-induced neuroinflammation by inhibiting Toll-like Receptor 4 (TLR4) signaling in the rat model without apparent cell replacement and differentiation [13, 20]. This suggests that the protective effects of MSCs may be mediated by paracrine signaling. Overall, the present study aimed to evaluate the neuroprotective and anti-neuroinflammatory effects and the possible mechanisms of MSC-EVs action in the OHT mice.

## Materials and methods

### Animals

Sixty male eight-week-old adult male Kun Ming (KM) mice were purchased from Slac Animal Co., Ltd. (Changsha, China). The number of animals involved in the study and their suffering were kept to a minimum. The Central South University’s Animal Care Ethics Committee authorized all animal experimental protocols. All animal experiments were complied with the regulations and guidelines established by the Animal Care and Use Committee of Central South University (Approval number: 2021sydw0136) and all experiments in this paper adhere to the ARRIVE guidelines. All animals were kept (four mice/cage) under standard laboratory conditions with a 12-hour light/dark cycle and standard humidity and temperature.

### EV production and characterization

Primary human ADSCs were procured from Cyagen Biosciences (Guangzhou, China) and cultured following the supplier’s guidelines. ADSCs were isolated from four healthy donors (information supported by the Cyagen Biosciences) and used from passages 3–5. Following the manufacturer’s instructions, adipogenic and osteogenic differentiation kits (Stem Cell Technologies, Canada) were used to perform adipogenic and osteogenic differentiation. Human antibodies labeled with fluorophore-conjugated were used to test CD44-PE, CD73-BV421, CD90-APC, CD105-PE, CD34-FITC, CD45-BV421 (BD Biosciences, USA) on the BD FACSCelesta flow cytometer. ADSCs were grown in medium without serum for 48 h after three PBS washes until they were 85–90% confluent. After three PBS washes, ADSCs were cultured in serum-free media for 48 h until they were 85–90% confluent. Then, to eliminate suspended cells and cell debris, the supernatants were collected and centrifuged for 15 min each at 400 g and 2,500 g at 4°C. The supernatants were then subjected to ultracentrifugation at 140,000 g at 4°C for 2 h (Beckman Coulter, USA) to pellet the EVs after filtration through a 0.8 μm membrane. The pellets containing ADSC-EVs were resuspended in DPBS for subsequent experiments. The phenotype of ADSC-EVs was examined using transmission electron microscopy (TEM), and the protein concentration was determined using a micro-BCA protein assay kit (Thermo Fisher Scientific, USA). The number of particles and their size distributions were measured by nanotracking analysis (ZetaView, Germany). Expression of marker proteins, CD9 and Alix (CST, USA) were detected by Western blotting.

### BV-2 cell culture and activation

BV-2 (microglial cell line) was obtained, cultured, and stimulated as described in our previous study. Briefly, complete media (DMEM + 10% FBS, Gibco, USA) was used to grow BV-2 cells at 37°C in a humidified incubator with 5% CO2. Lipopolysaccharide (LPS, 1 g/ml, Sigma, USA) was used to stimulate BV-2 cells for 24 h or LPS + ADSC-EVs (50 µg protein/ml) [21] for 24 h.

### ADSC-EVs labeling and cellular internalization

ADSC-EVs labeling was performed using 5µM CM-DiI Dye (Invitrogen, USA) for 30 min following the manufacturer’s instruction, followed by PBS washes and re-ultracentrifugation to remove the unbound dye. BV-2 cells were co-cultured with Dil-labeled ADSC-EVs for 10 h. The cytoskeleton and nuclei were stained with Alexa Fluor 488 phalloidin dye (CST, USA) and DAPI (CST, USA), respectively, before examination with a Zeiss confocal fluorescence microscope.

### RT‑qPCR

According to the manufacturer’s instructions, total RNA was extracted from BV-2 cells with the SteadyPure Quick Extraction Kit (Accurate Biotechnology, China). Complementary DNA (cDNA) was synthesized by RT SuperMix (Vazyme, China). The ChamQ Universal SYBR Mix (Vazyme, China) and LightCycler® 96 machine were used to conduct experiments and analyze data. The following primer sequences (5′-3′) were employed: mouse IL-6 (TCTGCAAGAGACTTCCATCCAGT, TCTGCAAGTGCATCATCGTTGT), mouse IL-1β (TCCTTGTGCAAGTGTCTGAAGC, ATGAGTGATACTGCCTGCCTGA), mouse TNF-α (GCCTCTTCTCATTCCTGCTT, CTCCTCCACTTGGTGGTTTG), mouse 18 S rRNA (GACTCAACACGGGAAACCTC, ATGCCAGAGTCTCGTTCGTT). Relative mRNA expression was obtained following the 2^−ΔΔCt^ method compared to 18 S rRNA. All measurements were performed in triplicate.

### Cytometric bead array assay (CBA)

The CBA kit (BD Biosciences, USA) was employed to quantify the concentrations of TNF-α, IL-6, and IL-1β in the BV-2 cell culture supernatant following the manufacturer’s recommendations. Measurements were performed by flow cytometry and analyzed using the CBA software (FCAP Array).

### Induction of OHT, IOP measurement, and ADSC-EV administration

The OHT mouse model was established as described in our previous study [13, 20]. Specifically, the animals were anesthetized by intraperitoneal injection of 1.25% tribromoethanol (20 µl/g, Lab Anim Sci-Tech, China) for dilating pupils and anesthetic eye drops with tropicamide phenylephrine (Santen, Japan) and oxybuprocaine hydrochloride (Santen, Japan). After anterior chamber penetration, we injected 2 µL air and 3 µL microsphere suspension (1 × 10^5^ microspheres/µL, 10 μm diameter, Thermo Fisher Scientific, USA) into the anterior chamber to establish the OHT model. Using a handheld tonometer (TonoLab, Finland), IOP was measured once every six days for 17 days at approximately the same time during at the 9 AM and 11 AM. Six measurements from each eye were recorded and averaged. To explore the protective effects of ADSC-EVs, KM mice were split into the following categories at random (*n* = 20): Control: sham; OHT + ADSC-EVs: OHT mice treated with intravitreal injection of 3 µg (4.5 × 10^8^ particles) ADSC-EVs in 1.5 µl PBS, and OHT + PBS: OHT mice treated with intravitreal injection of 1.5 µl PBS. ADSC-EVs and PBS were administered intravitreally as described in our previous study by using a 33G Hamilton syringe (Hamilton, USA).

### Tissue preparation, histological analysis, and immunohistochemistry

Optic nerve and eyeball tissues were harvested, fixed in 3% paraformaldehyde (PFA, Solarbio, China) at 4°C for 4 h, and then either dissected for retinal flatmounts or for tissue slices. Retinal flatmounts and 10 μm slices were permeabilized and blocked in 0.4% Triton X-100 and blocking buffer at room temperature (RT) for two hours. Subsequently, the samples were incubated with primary antibodies (1:100, Novus, USA) overnight at 4°C. The following primary antibodies were used: goat anti-Iba-1 antibody, rat anti-CD68 antibody, mouse anti-CCL2 antibody, and mouse anti-TLR4 antibody. After washing, the samples were incubated with species-specific secondary antibodies at RT for 60 min, rewashed, and counterstained with DAPI (Vector Laboratories, USA). To evaluate RGC axonal loss, we obtained semithin transverse sections from the optic nerve, 2 mm distal to the globe. These sections were stained with 1% toluidine blue and then photographed.

### Optical coherence tomography (OCT)

OCT was used to measure inner retinal thickness as previously described [20]. Briefly, fundus and OCT images of the retina around the optic disc were obtained, and the Insight software (Phoenix Research Labs, USA) was used to measure the retinal thickness.

### Visual evoked potential (VEP)

Flash VEP (Roland, Germany) examination was used to record RGC function. Mice were dark-adapted for more than 10 h and anesthetized as before. After pupil dilation, needle electrodes were placed under the nasal skin and subdermally in the midline of the head, respectively. Mice were then exposed to flashes of 1 Hz, 3 cd·s/m^2^. The amplitude and latency of P1 waves were used.

### Microscopy

All fluorescently stained images were taken using a Zeiss confocal fluorescence microscope with stable settings. For retinal flat-mounted Iba-1^+^ cells, images were taken at ×20 magnification with z-stack. Two independent, blinded researchers used ImageJ to count Iba-1^+^ cells in five retinal pictures. The RBPMS^+^ RGC counting was performed using the same method. For Iba-1^+^ cells in the optic nerve cross-section, images were taken at ×20 magnification. We quantified the expression of CD68, Iba-1, CCL2, and TLR4 in the retina and optic nerve sections by measuring their fluorescence intensity using the ImageJ software.

### Western blot analysis

RIPA lysis buffer including protease inhibitors (Sigma, USA) was used to obtain the total protein from ADSC-EVs, BV-2 cells, retinas, and optic nerves. Pierce BCA assay (Thermo Scientific, USA) was used to quantify the protein amount. Proteins (30 µg) were separated by SDS-PAGE and transferred onto PVDF membranes (Millipore, USA). Before primary antibody incubation, the membranes were blocked using QuickBlock™ buffer (Beyotime Biotechnology, China) at RT for 20 min. The following primary antibodies (1:800, CST, USA) were used: mouse anti-Alix antibody, rabbit anti-CD9 antibody, mouse anti-GAPDH, rabbit anti-TNF-α, rabbit anti-IL-6, rabbit anti-IL-1β, rabbit anti-β-actin, mouse anti-TLR4 antibody, rabbit anti-phospho-Erk, rabbit anti-phospho-p38 MAPK, and rabbit anti-phospho-NF-κB p65. The PVDF membranes were then incubated for 60 min at RT using species-specific secondary antibodies, detected using the enhanced chemiluminescence (ECL, Millipore, USA) solution, and captured on the Odyssey Imaging System (LI-COR, USA).

### Statistical analysis

We used the Prism 8 software to analyze all the data. The mean and standard error of the mean were computed to express our results. Student’s t-test or one-way ANOVA followed by Tukey’s multiple comparison test were used to assess the statistical differences.

## Results

### OHT induces retinal and optic nerve microglial proliferation, activation, and neuroinflammatory response in mouse models

**Fig. 1:**
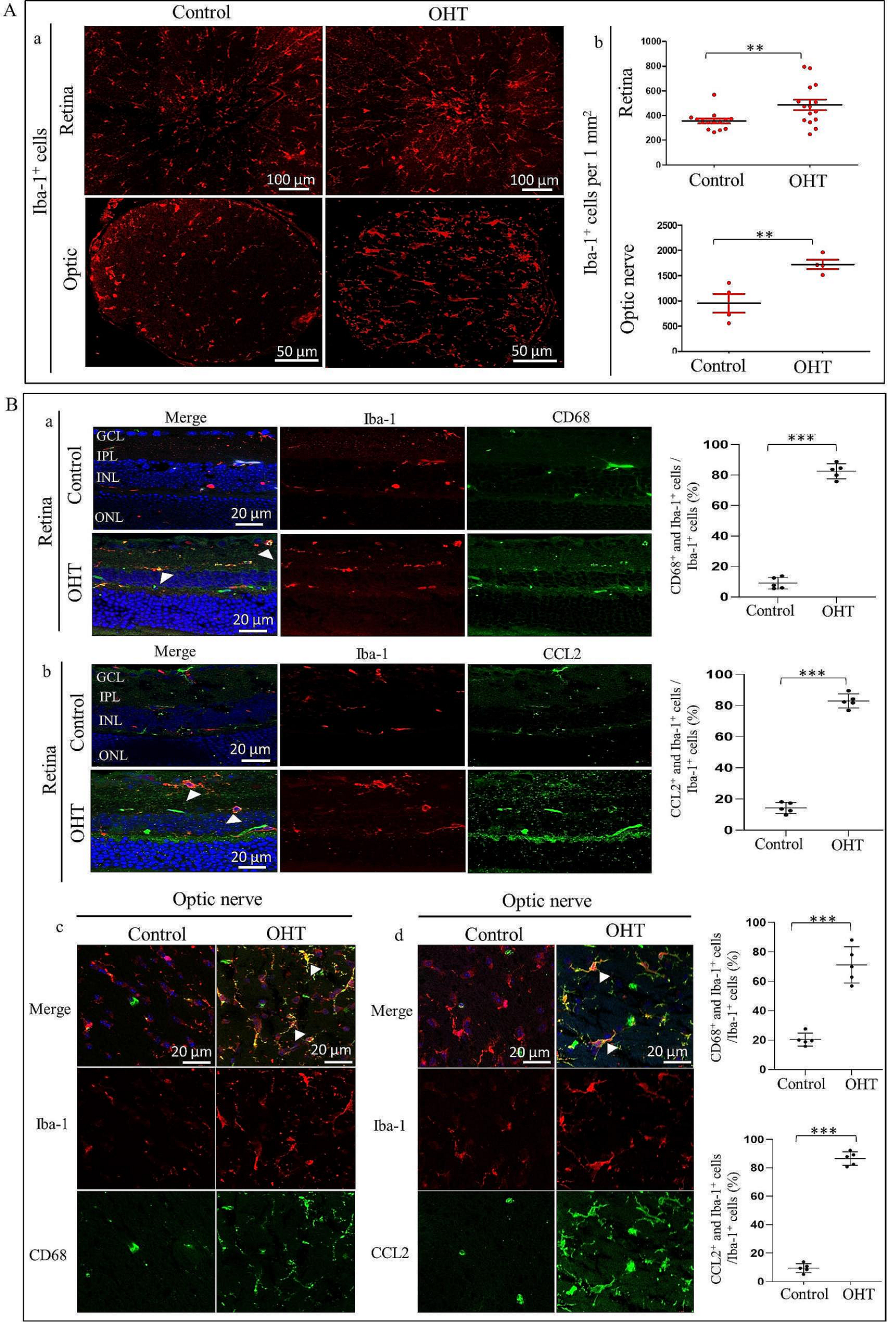
Activation and proliferation of microglial cells in OHT mice. (**A**) Increased Iba-1 expression in microglia from OHT mice. (a) Iba-1 immunostaining (red) in the retina and optic nerve of intracameral injection of microsphere-induced OHT mice and PBS control. (b) Quantification of Iba-1^+^ microglia cells. (*n* = 3–4, **p < 0.01). (**B**) Increased CD68 and CCL2 expressions in microglia of OHT mice. (a, b) Immunostaining images of the retina from control and OHT mice at 7 days, demonstrated that activated microglia in the OHT group had a strong co-expression of CD68 or CCL2 with Iba-1 positive microglia (white arrows, *n* = 5, ***p < 0.001). (c, d) Representative immunostaining images of the optic nerve in control and OHT mice at 7 days, showed that activated microglia in the OHT group had a strong co-expression of CD68 or CCL2 with Iba-1 positive microglia (white arrows, *n* = 5, ***p < 0.001). GCL, ganglion cell layer; IPL, inner plexiform layer; INL, inner nuclear layer; ONL, outer nuclear layer

To investigate the involvement of microglia, we examined the number and expression of CD68 (a marker of microglial activation) and CCL2 (an immuno-inflammatory response chemokine) [22] in the proximal optic nerve and retina of OHT mice after one week of anterior chamber microsphere injection. Immunofluorescence images of the optic nerve and retina flat mount cross section showed that compared to the control eyes with PBS anterior chamber injection, the amount of Iba-1^+^ microglia in OHT mice was significantly higher (Fig. 1A). Double immunofluorescence images of optic nerve section and retina showed that Iba-1^+^/CD68^+^ and Iba-1^+^/CCL2^+^ microglia were markedly increased compared to the PBS-injected eyes (Fig. 1B). Our data suggest that microglia are involved in the OHT models through proliferation, activation, and neuroinflammation.

### OHT induces retinal and optic nerve macroglia (astrocytes and Müller cells) activation in OHT mice

Since the macroglia also contribute to neuroinflammation, we examined the expression of GFAP and CCL2 in the optic nerve and retina of OHT mice after one week after anterior chamber microsphere injection (Fig. S1). Double immunofluorescence images of optic nerve section and retina GFAP (green) and CCL2 (red) showed a clear increase in CCL2 and GFAP expression in the OHT group compared to the control group, suggesting activation of astrocytes, but without a clear overlap between CCL2 and GFAP. Interestingly, OHT promotes increased co-localization of GFAP and CCL2, but most of the expression of CCL2 seems to occur mainly outside of GFAP. Therefore, in our present study, we decided to focus first on the microglia-induced neuroinflammation.

### Characterization of human ADSCs and ADSC-EVs

**Fig. 2:**
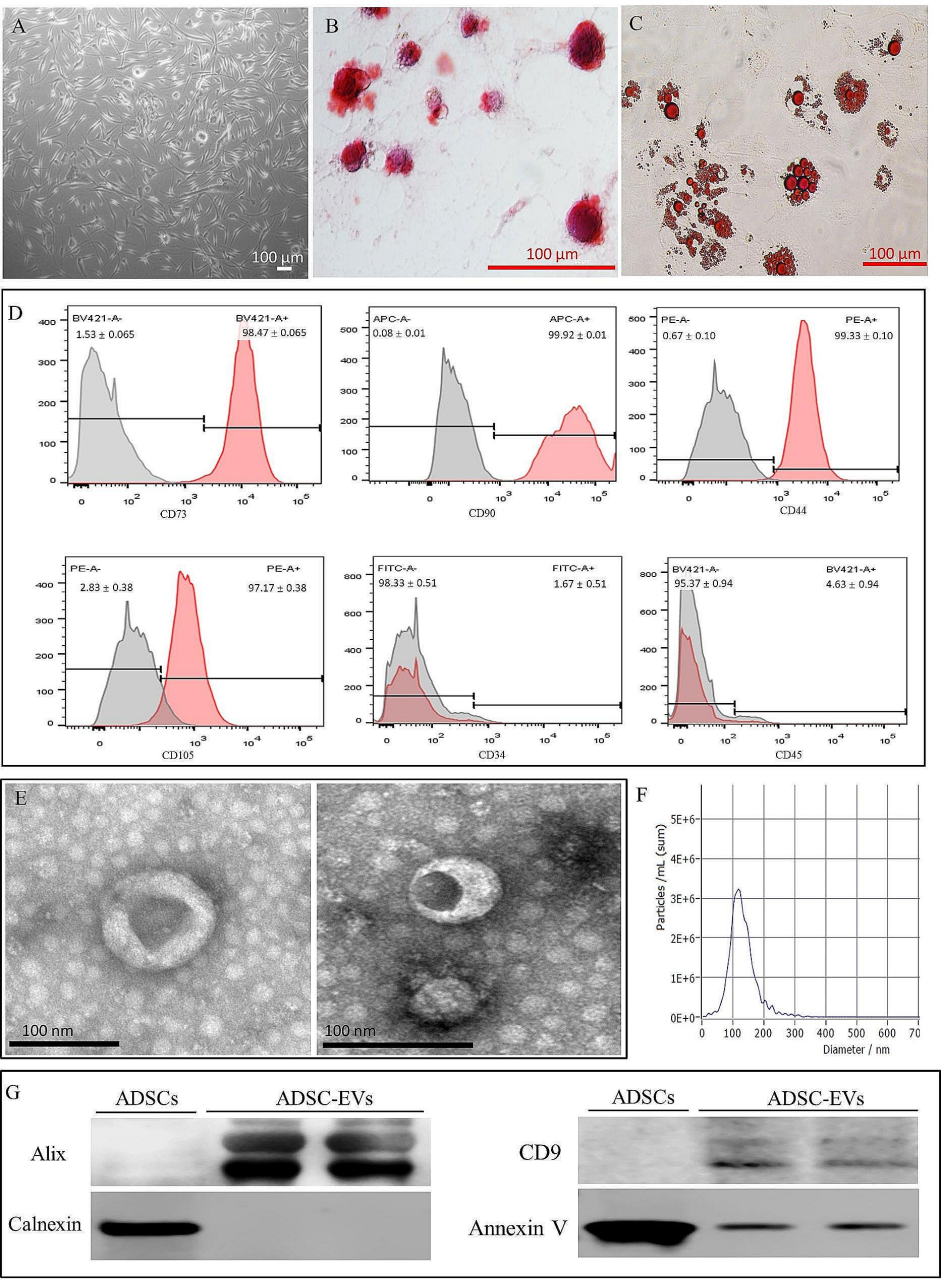
Characterization of human ADSCs and ADSC-EVs. (**A**) Representative morphology of ADSCs. (**B**) Representative osteogenic differentiation. (**C**) Representative adipogenic differentiation. (**D**) Results of flow cytometry histograms of ADSCs. (**E**) Representative transmission electron microscopy (TEM) images of ADSC-EVs. (**F**) Nanoparticle tracking analysis (NTA) histogram image of ADSC-EVs. (**G**) Representative Western blotting images of ADSC-EVs

Human ADSCs showed a fibroblast-like morphology (Fig. 2A). In conditioned media, alizarin red and oil-red O staining demonstrated that ADSCs could differentiate into osteocytes and adipocytes (Fig. 2B and C). The results of flow cytometry suggested that ADSCs had low CD34 and CD45 markers expression and extremely high CD73, CD90, CD44, and CD105 markers expression. ADSC-EVs were identified. TEM revealed that ADSC-EVs had a characteristic cup-shaped membrane structure with two layers (Fig. 2E). NTA (Fig. 2F) showed that the diameter of ADSC-EVs ranged from 30 to 200 nm with a peak at 116.8 nm, and the concentration was approximately 1.1 × 10^10^ particles/ml. ADSC secreted 2.75 × 10^8^ particles EVs/100,000 cells/48 h. Results from western blotting revealed that ADSC-EVs were negative for the GAPDH and positive for specific markers including CD9 and Alix (Fig. 2G). These data indicate that ADSC-EVs possess the characteristics of EVs.

### ADSC-EVs treatment decreases LPS-induced microglia activation and TLR4/NF-κB proinflammatory cascade signaling in vitro

**Fig. 3:**
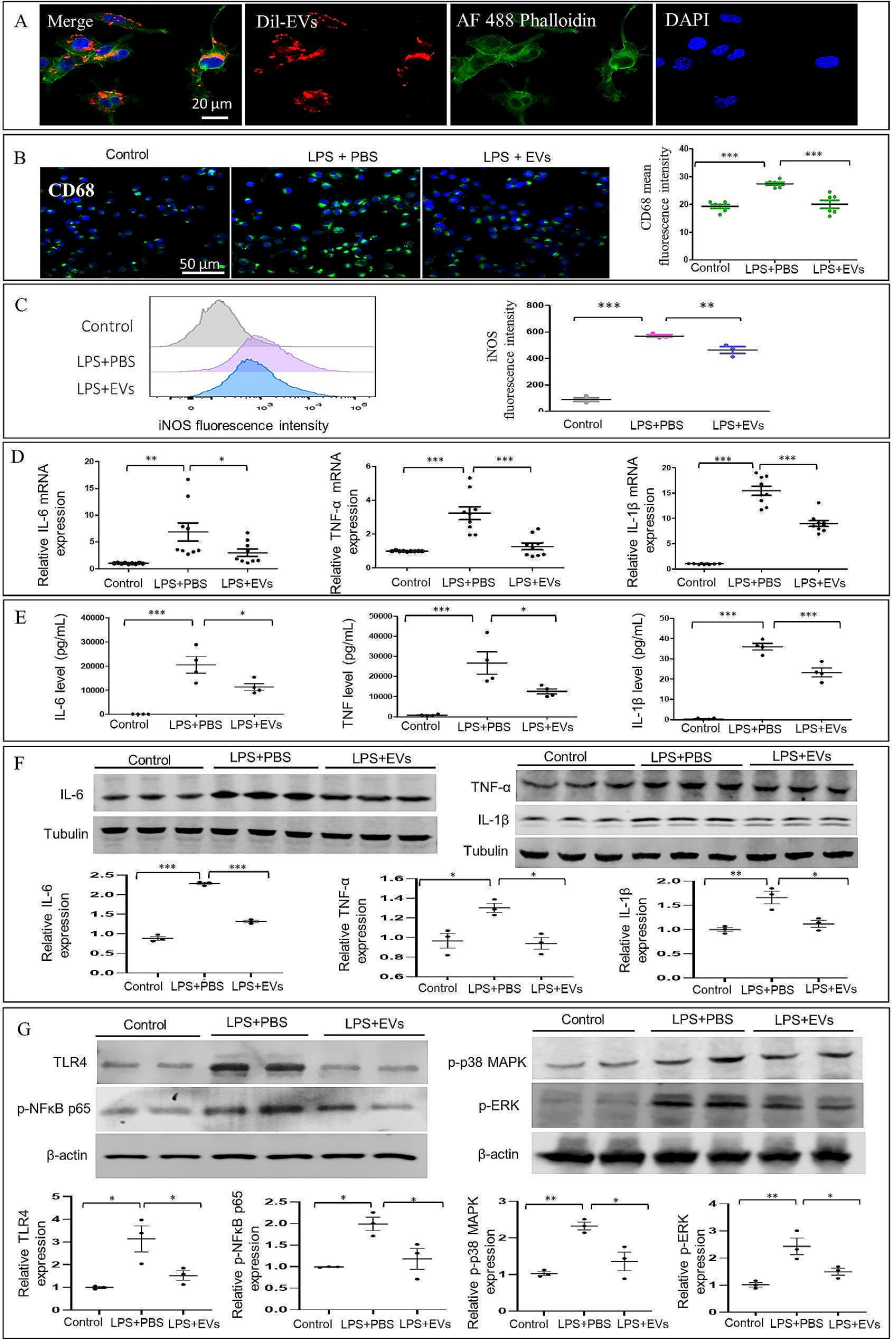
ADSC-EVs decrease LPS-stimulated activation, TLR4/MAPK/NF-κB proinflammatory cascade signaling in BV2 cells induced by LPS. (**A**) Representative images of the uptake of Dil-labeled EVs (red) by BV2 cells. (**B**) Representative immunostaining images and quantification of CD68 in LPS-activated BV2 cells after culturing with PBS or ADSC-EVs for 24 h (*n* = 6, ***p < 0.001). (**C**) Representative flow cytometry images and iNOS fluorescence intensity quantification in LPS-activated BV2 cells after culturing with PBS or ADSC-EVs for 24 h (*n* = 3, **p < 0.01, ***p < 0.001). (**D**) Quantification of IL-1β, IL-6, and TNF-α mRNA levels relative to 18s RNA by qRT-PCR in LPS-activated BV2 cells after culture with PBS or ADSC-EVs for 24 h (*n* = 3, *p < 0.05, **p < 0.01, ***p < 0.001). (**E**) Quantification of IL-1β, IL-6, and TNF-α production in LPS-stimulated BV2 cell culture medium after treatment with ADSC-EVs or PBS for 24 h (*n* = 4, *p < 0.05, ***p < 0.001). (**F**) Western blot images of IL-6, TNF-α, and IL-1β relative protein expression normalized to tubulin levels (*n* = 3, *p < 0.05, **p < 0.01, ***p < 0.001). (**G**) Western blot images of TLR4, p-NFκB, p65, p-p38 MAPK, and p-ERK relative protein expression normalized to Western blot images of IL-6, TNF-α, and IL-1β relative protein expression normalized to tubulin levels (*n* = 3, *p < 0.05, **p < 0.01, ***p < 0.001)

We confirmed the cellular uptake of ADSC-EVs by BV-2 cells before examining the impact of ADSC-EVs on the activation and proinflammatory response of BV-2 microglia. By labeling ADSC-EVs with CM-DiI dye, they were cocultured with BV-2 cells for 10 h. The results indicated that ADSC-EVs could be internalized by BV-2 (Fig. 3A). Next, BV-2 cells were stimulated using 1 µg/ml LPS + 50 µg/ml ADSC-EVs or PBS for 24 h. Cell immunofluorescence staining and flow cytometry analysis showed that ADSC-EVs significantly reduced the expression of activation markers (CD68 and iNOS) of microglial cells (Fig. 3B-C). To determine the impact of ADSC-EVs on the proinflammatory response, the mRNA and protein expressions of the proinflammatory cytokines, IL-6, IL-1β, and TNF-α, were evaluated by CBA kit, RT-qPCR, and Western blotting. Compared to the LPS + PBS cell group, ADSC-EVs significantly decreased proinflammatory cytokine expression (Fig. 3D-F). Studies have reported that NF-κB phosphorylation regulates the production of proinflammatory cytokines [23]; additionally, our previous work showed that MSCs inhibited proinflammatory cytokines and the TLR4 pathway in LPS-stimulated BV-2 cells. Therefore, we next investigated if ADSC-EVs affected the TLR4/MAPK/NF-κB signaling pathway. Compared to the LPS + PBS cell group, western blot analysis revealed that the protein expressions of TLR4, p-NFκB p65, p-p38 MAPK, and p-Erk were significantly reduced by ADSC-EVs. Taken together, our findings showed that ADSC-EV treatment decreases microglia activation and TLR4/MAPK/NF-κB proinflammatory cascade in vitro.

### ADSC-EVs treatment inhibits further RGC damage from ocular hypertension

**Fig. 4:**
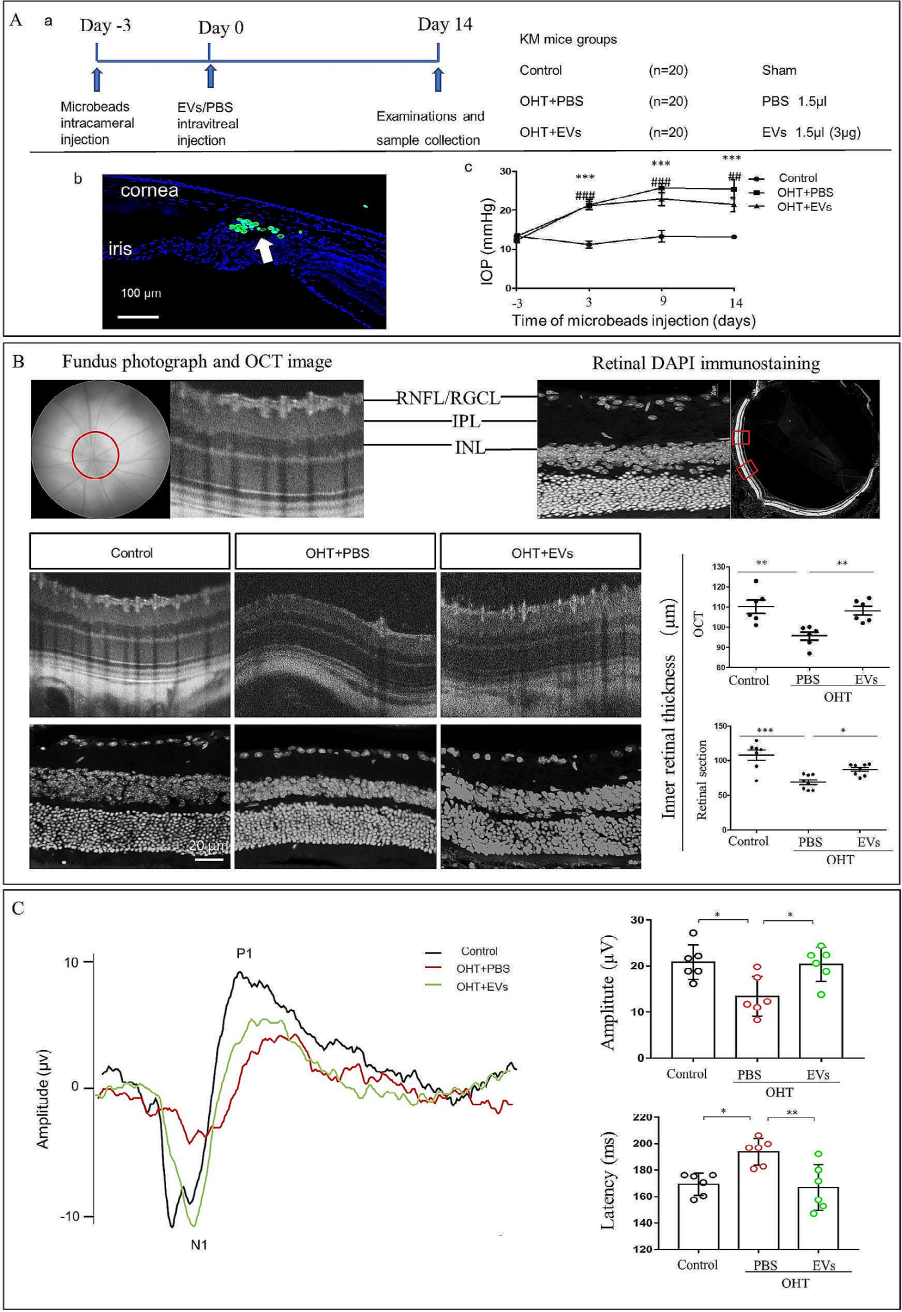
ADSC-EVs promote inner retinal thickness and retinal function in the OHT mice 2 weeks after injection. (**A**) In vivo experimental design. (a) Timeline of the study design when microbeads injection, intravitreal administration, and sample collection were carried out. (b) Representative fluorescence image of the anterior segment cross-Sect. 14 days after microbeads (arrow) injection. (c) Simultaneous IOP measurements in OHT eyes compared to the control group. ***p < 0.001 OHT + PBS eyes vs. control. ^###^*p* < 0.001 OHT + EVs eyes vs. control, ^##^*p* < 0.01 OHT + EVs eyes vs. control. (**B**) Representative fundus photographs, OCT images, retinal DAPI immunostaining and quantification of inner retinal thickness (*n* = 6–9, **p < 0.01, ***p < 0.001). RNFL (retinal nerve fiber layer), RGCL (retinal ganglion cell layer), IPL (inner plexiform layer), INL (inner nuclear layer). (**C**) F-VEP examination results in per group. (*n* = 6, ***p < 0.001)

**Fig. 5:**
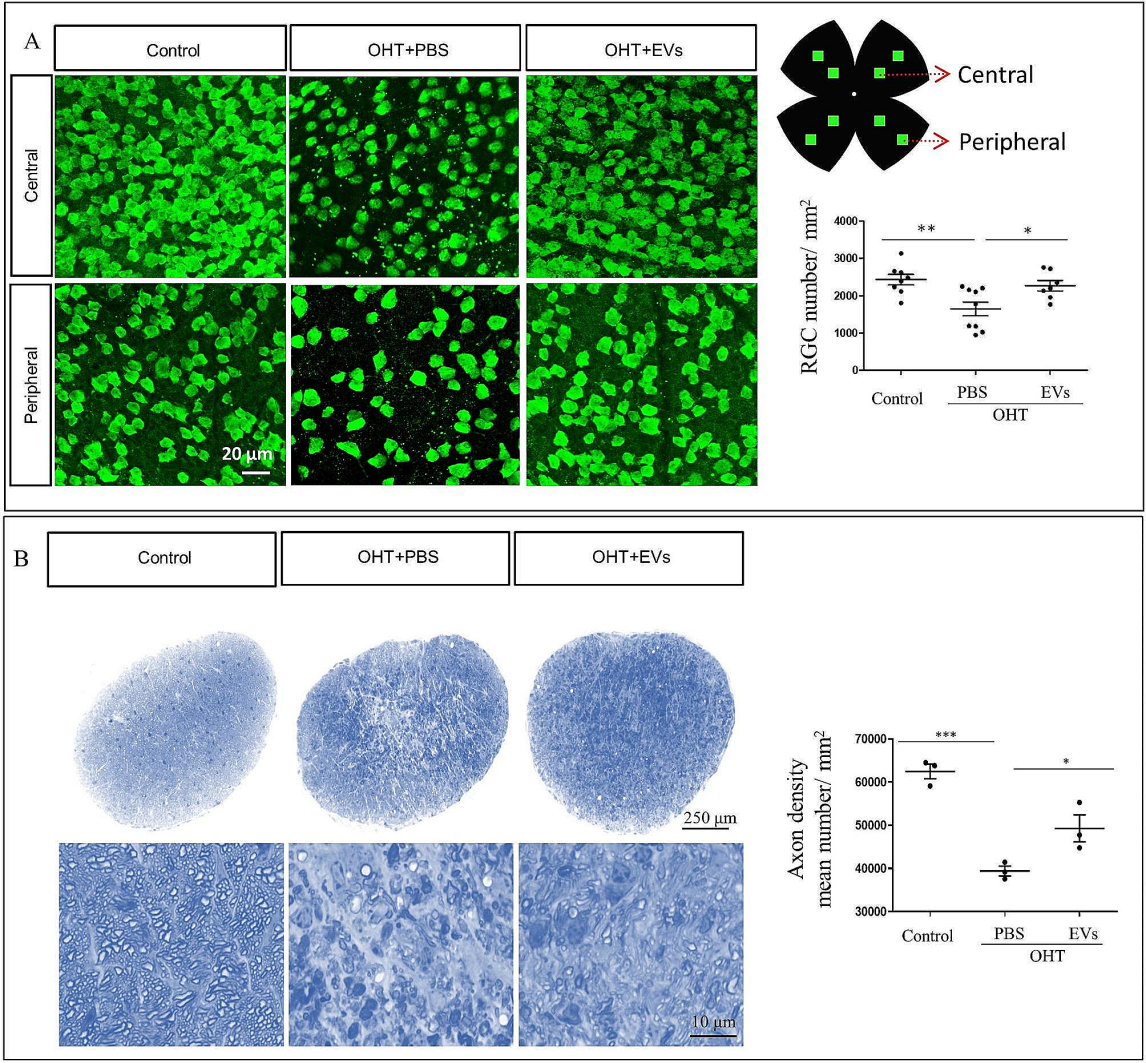
ADSC-EVs prevent further RGC and axon loss in OHT mice 2 weeks after injection. (**A**) Representative retinal immunostaining and quantification of the RBPMS^+^ RGCs (*n* = 8, *p < 0.05, **p < 0.01). (**B**) Representative optic nerve semithin transverse sections and quantification of axons (*n* = 3, *p < 0.05, ***p < 0.001)

We designed the in vivo experiment as shown in Fig. 4A to investigate the effect of ADSC-EVs using OHT mice. We evaluated the inner retinal thickness and RGC function. The OCT results showed that after 2 weeks of intravitreal injection of ADSC-EVs in the OHT mice, the inner retinal thickness was thicker (108.2 ± 2.22 μm) than in the PBS injection groups (95.58 ± 2.039 μm). Similarly, retinal sections quantification results showed that the inner retinal thickness in ADSC-EVs injection mice was thicker (87.38 ± 2.872 μm) than in the PBS injection groups (69.00 ± 3.640 μm; Fig. 4B). The flash-VEP was used to examine the RGC function, our results demonstrated that the P1 amplitude in ADSC-EVs treatment group (20.38 ± 1.53 µV) was significantly higher than that in the PBS group (13.43 ± 1.77 µV); and the P1 latency in ADSC-EVs treatment group (193.9 ± 4.06 ms) was significantly lower than that in the PBS group (166.9 ± 7.09 ms; Fig. 4C). Meanwhile, the positive immunofluorescence of RBPMS RGCs counts were significantly increased after ADSC-EVs treatment, the number of RGCs was increased from 1646 ± 184.8/mm^2^ in the PBS + OHT group to 2268 ± 139.9/mm^2^ in the ADSC-EVs + OHT group (Fig. 5A). The RGCs axon loss in the ADSC-EVs injection group was also decreased, the axon number was increased from 39,412 ± 1123/mm^2^ in the PBS + OHT group to 49,275 ± 3127/mm^2^ in the ADSC-EVs + OHT group (Fig. 5B). These results indicated that ADSC-EVs treatment inhibits further RGC damage from ocular hypertension.

### ADSC-EVs treatment decreases microglia activation and neuroinflammatory response chemokines in the retina and optic nerve of OHT animals

**Fig. 6:**
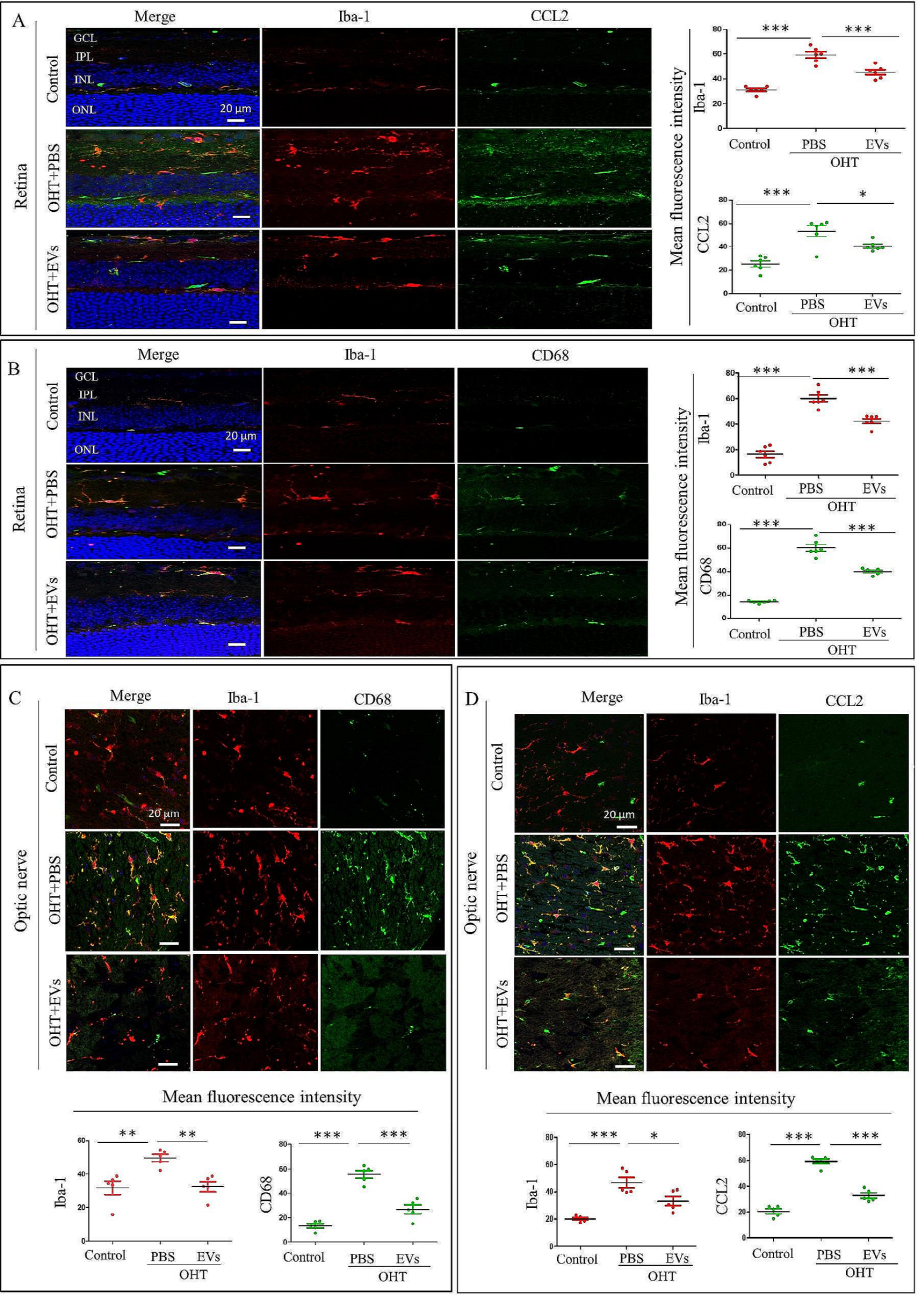
ADSC-EVs reduce microglial activation and microglia-derived neuroinflammatory response chemokines in the retina and optic nerve of OHT mice 2 weeks after injection. (**A**) Representative retinal immunostaining and quantification of the expression of CCL2 and Iba-1 (*n* = 5, **p < 0.01, ***p < 0.001). (**B**) Representative retina immunostaining and quantification of the expression of CD68 and Iba-1 (*n* = 6, *p < 0.05, ***p < 0.001). (**C**) Representative optic nerve immunostaining and quantification of the expression of CD68 and Iba-1 (*n* = 5, **p < 0.01, ***p < 0.001). (**D**) Representative optic nerve immunostaining and quantification of the expression of CCL2 and Iba-1 (*n* = 5, *p < 0.05, ***p < 0.001)

Iba-1 immunostaining showed ramified microglia located in the plexiform layers of the retina and throughout the optic nerve cross-section. Fourteen days after ADSC-EVs intravitreal injection, Iba-1/CD68 double immunostaining was used to analyze microglial activation. In the control groups, faint CD68 immunosignal was observed in both optic nerve and retina. In the OHT + PBS group, an increased fluorescence intensity of CD68 and Iba-1 microglia was detected, while in the ADSC-EVs group, Iba-1 and CD68 showed a significantly decreased fluorescence signal (Fig. 6B-C). Similarly, the analysis of microglia-related neuroinflammatory response chemokines was performed by Iba-1/CCL2 double immunostaining in the retina (Fig. 6A) and optic nerve (Fig. 6D). In ADSC-EVs-treated OHT mice, Iba-1 and CD68 fluorescence signals were significantly reduced compared with PBS treatment both in optic nerve and retina. Taken together, our findings indicated that ADSC-EV administration decreases microglia activation and neuroinflammatory response chemokines in the retina and optic nerve of OHT animals.

### ADSC-EV treatment inhibits microglia-associated TLR4 and TLR4/MAPK/NF-κB proinflammatory cascade in the retina and optic nerve of OHT animals

**Fig. 7:**
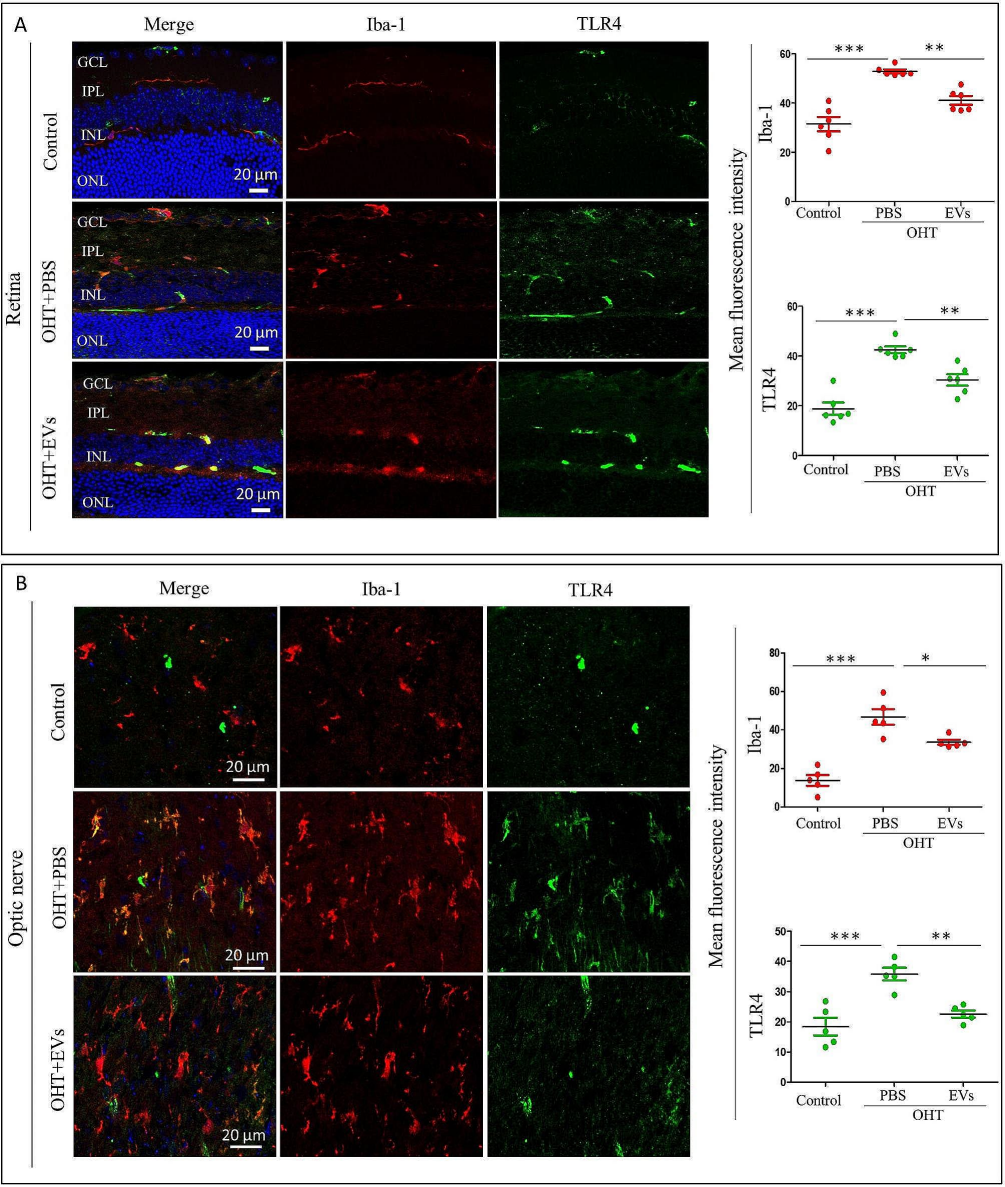
ADSC-EVs decrease microglia-associated TLR4 expression in the OHT mice 2 weeks after injection. (**A**) Representative retina immunostaining and quantification of the expression of Iba-1 and TLR4 (*n* = 6, **p < 0.01, ***p < 0.001). (**B**) Representative optic nerve immunostaining and quantification of the expression of TLR4 and Iba-1 (*n* = 5, *p < 0.05, **p < 0.01, ***p < 0.001)

**Fig. 8:**
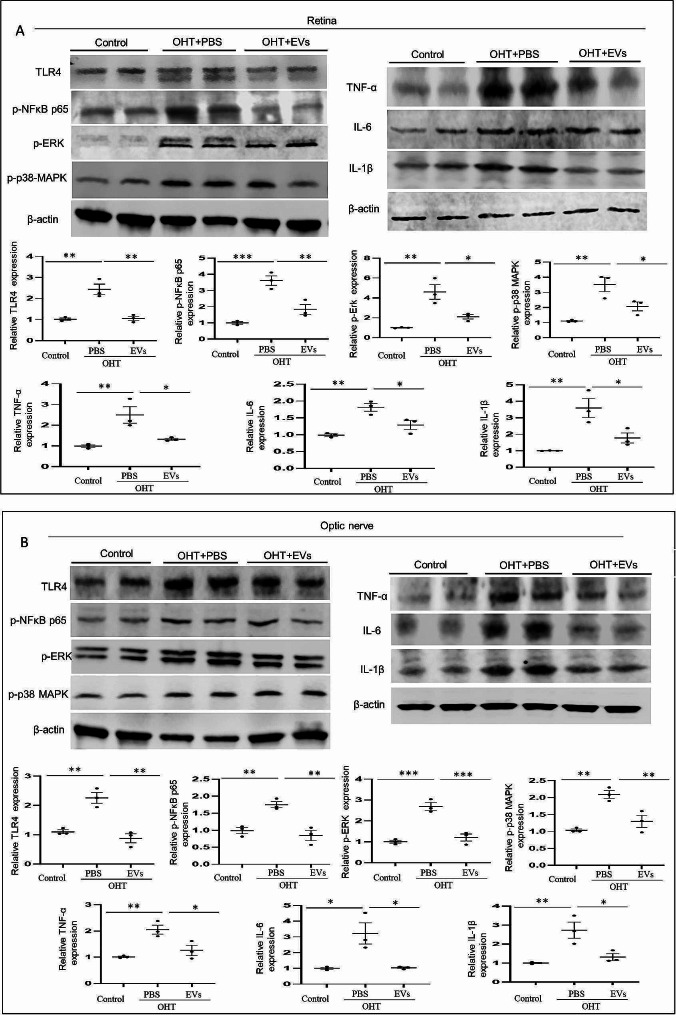
ADSC-EVs suppress OHT-induced TLR4/MAPK/NF-κB proinflammatory cascade signaling in the OHT mice 2 weeks after injection. (**A**) Western blot images of TLR4, p-p38 MAPK, p-NFκB, p65, p-ERK, IL-6, TNF-α, and IL-1β relative protein expressions normalized to β-actin levels in the retina (*n* = 3, *p < 0.05, **p < 0.01, ***p < 0.001). (**B**) Western blot images of TLR4, p-p38 MAPK, p-NFκB, p65, p-ERK, IL-6, TNF-α, and IL-1β relative protein expressions normalized to β-actin levels in the optic nerve (*n* = 3, *p < 0.05, **p < 0.01)

We next determined the effects of ADSC-EVs associated with TLR4, TLR4/ p-38 MAPK/NF-κB inflammatory cascade in vivo using OHT mice fourteen days after ADSC-EVs intravitreal injection. We investigated the level of microglial TLR4 in the retina and optic nerve (Fig. 7A-B) by TLR4/Iba-1 double immunostaining. In ADSC-EVs-treated OHT mice, Iba-1 and TLR4 fluorescence signals were significantly reduced compared with PBS-treated OHT mice, both in the retina and optic nerve. We then examined whether ADSC-EVs could affect TLR4/MAPK/NF-κB proinflammatory cascade in the OHT mice in the retina and optic nerve. According to the Western blot data ADSC-EVs treatment reduced the protein levels of TLR4, p-NFκB p65, p-p38 MAPK, p-Erk, IL-1β, TNF-α, and IL-6, compared to those in PBS-treated OHT mice in the retina and optic nerve (Fig. 8A-B). Taken together, these results indicated that ADSC-EVs treatment inhibits microglia and mechanistically, this is associated with TLR4 and TLR4/MAPK/NF-κB proinflammatory cascade.

## Discussion

In our previous studies, we showed that MSCs have neuroprotective and anti-neuroinflammatory effects through the modulation of microglia activation and inhibition of the TLR4 signaling in the microbead injection-induced OHT animal models without apparent MSC-related differentiation and replacement. Therefore, we hypothesized that the above protective effects of MSCs might be due to their secretory function. In this work, we present evidence that intravitreal treatment of ADSC-EVs could attenuate OHT-induced RGC damage and microglial neuroinflammatory responses by reducing TLR4/MAPK/NF-κB proinflammatory signaling, resulting in the decreased levels of proinflammatory cytokines.

Previous studies suggest that microglial activation and microglia-associated neuroinflammation are involved in the IOP elevation related to retinal and optic nerve injury both in animal models and patients with glaucoma [2, 24, 25]. Microglial activation is a complex pathophysiological phenomenon including soma shape transformation, density change, and expression of activation markers including MHCII and CD68 [25, 26]. As one of the primary chemokines influencing microglial movement and luring cells engaged in the immune/inflammatory responses, the expression of C-C motif ligand 2 (CCL2), also known as monocytic chemotactic protein 1 (MCP-1), also known as monocyte chemoattractant protein-1 (MCP‐1), was examined to determine the microglia as one of the sites of neuroinflammatory responses. In our present study, we found increased cell density, co-expression of Iba-1 with the activation marker CD68, and the neuroinflammatory response maker CCL2, and morphologic changes in microglia within 7 days after the microbeads intracameral injection before the early RGCs degenerative changes occur [27]. Results from our animal models indicate that microglial cells are involved in OHT through proliferation, activation, and neuroinflammation, consistent with studies using similar and other high IOP animal models [28].

Chronic neuroinflammation is involved in retinal and optic nerve injury in animals with elevated IOP [8, 28]. Our previous study indicated that activated microglia contribute to chronic neuroinflammation through the activation of the TLR4 pathway. TLR4 activation then promotes proinflammatory cytokines, resulting in a chronic neuroinflammatory environment. Therefore, strategies that inhibit proinflammation caused by microglia have received more attention. Over the past decade, MSCs have been reported in the treatment of retinal and optic nerve injury, mostly through paracrine actions [29, 30]. Recently, MSC-EVs have been studied as a biomimetic alternative for MSCs but with lower oncogenic and immunological risks [31]. MSC-EVs can be used to treat brain neuroinflammation and other neurodegenerative diseases [32, 33]. Compared with MSC-EVs from other sources, ADSC-EVs have a better role in immunoinflammatory regulation [34]. Our study first investigated the protective function of ADSC-EVs on RGCs, and then evaluated the effects of ADSC-EVs on microglial activation marker, CD68, neuroinflammatory response maker, CCL2, and TLR4, p-NFκB p65, p-p38 MAPK, p-ERK, IL-1β, IL-6, and TNF-α.

We used the immortalized BV2 cell microglia to perform our in vitro experiments because 90% of the genes between BV-2 cells and primary microglia are shared [27, 35, 36]. Our results demonstrated that the ADSC-EVs could be internalized by BV-2 cells, which is a necessary process for their protective effect. In the LPS-stimulated BV-2 cells, ADSC-EVs could reduce the expression of microglia activation makers, CD68 and INOS. Next, we demonstrated that ADSC-EVs could diminish the gene and protein levels of IL-6, IL-1β, and TNF-α. Our data are consistent with previous findings that human Wharton’s jelly MSC-EVs dampened the mRNA levels of proinflammatory cytokines in BV-2 cells and primary microglial cells [37]. In line with this, Kodali, M. et al. [38] demonstrated that the human bone marrow MSC-EVs reduced the mRNA levels of IL-6 in mouse macrophages stimulated with LPS. These findings and our data together imply that ADSC-EVs are effective in decreasing proinflammation. Since the phosphorylation of NF-κB regulates the production of proinflammatory cytokines [23], combined with our previous findings that MSCs inhibit proinflammatory cytokine levels and TLR4 pathway in LPS-stimulated BV-2 cells, we further investigated the NF-κB signaling in detail. Here, we demonstrated that ADSC-EV treatment decreased microglial-associated neuroinflammatory responses by interfering with the TLR4/p-38 MAPK/NF-κB inflammatory cascade, including TLR4, p-NFκB p65, p-p38 MAPK, p-ERK. Notably, BV-2 is an immortalized cell line that can take up ADSC-EVs and respond to LPS. Further studies using primary microglial cells may be required to confirm the results and mechanism identified here. ADSC-EVs could attenuate LPS-activated microglial TLR4/MAPK/NF-κB proinflammatory cascade signaling in vitro. These results encourage the investigation of the anti-neuroinflammatory effect of ADSC-EVs in vivo in rodent models.

Here we demonstrated that intravitreal administration of ADSC-EVs significantly reversed the RGC function (F-VEP) and RGC damage (inner retinal thickness) in OHT mice. This significant RGC neuroprotection is consistent with the inhibition of further damage effects of RGCs (both soma and axon) in OHT mice, which is corroborated by other studies showing the RGC neuroprotection of MSC-derived EVs in various glaucoma rodent models [39–42]. Although different sources of MSC-EVs have been reported to have neuroprotective effects, the underlying mechanisms are not the same and a systemic comparison should be made. OHT mice had activated microglia and showed microglia-associated neuroinflammatory responses. ADSC-EVs could decrease the microglia-associated CD68, CCL2, and TLR4 expressions in OHT mice retina and optic nerve, consistent with results in other animal models showing the inhibitory effect of MSC-EVs on the TLR4 pathway [23, 37, 43]. These findings are especially important for exploring the mechanism underlying ADSC-EVs’ protective action in the OHT retina and optic nerve. We then investigated the TLR4/MAPK/NF-κB proinflammatory cascade pathway in OHT mice retina and optic nerve. ADSC-EVs robustly attenuated the TLR4/p-38 MAPK/NF-κB inflammatory cascade in the optic nerve and retina. Similar results were supported by studies demonstrating that MSC-EVs ameliorated microglia-mediated proinflammation in other diseases [25, 37, 43]. EVs contain mRNAs, miRNAs, lipids, and proteins, and the exact molecules that exert the corresponding effects need further exploration. Considering microglia-mediated neuroinflammation is involved in various neurodegenerative diseases including diabetic retinopathy [44], glaucoma [28], and age-related macular degeneration [45], ADSC-EVs modulating microglia should be a primary treatment strategy. In addition, how to employ ADSC-EVs to function specifically and in a targeted manner is challenging and needs to be addressed in further studies.

## Conclusions

In this study, we highlight an important function of ADSC-EVs in attenuating RGC damage and microglia-associated neuroinflammatory responses by reducing the TLR4/MAPK/NF-κB proinflammatory cascade in OHT mice. Since intravitreal administration of ADSC-EVs did not show any direct adverse effects, they are potential treatment candidates or adjunctive therapeutic options for glaucoma due to their anti-neuroinflammatory effects.

### Electronic supplementary material

Below is the link to the electronic supplementary material.

Supplementary Material 1

## Data Availability

The data used in this study are available from the corresponding author upon reasonable request.

## Abbreviations

ADSC-EVs: Adipose tissue-derived stem cell extracellular vesicles
CCL2: Chemokine (C-C Motif) Ligand 2
DMEM: Dulbecco’s modified eagle medium
EGF: Epithelial growth factor
FBS: Fetal bovine serum
FGF: Fibroblast growth factor
IOP: Intraocular pressure
IL: Interleukin
IPL: Inner plexiform layer
LPS: Lipopolysaccharide
MAPK: Mitogen-activated protein kinase
MSC: Mesenchymal stem cells
OHT: Ocular hypertension
PBS: Phosphate-buffered saline
PFA: paraformaldehyde
RGC: Retinal ganglion cell
RNFL: Retinal nerve fiber layer
TNF-α: Tumor necrosis factor α
RT-PCR: Quantitative real‑time polymerase chain reaction
NF-κB: Nuclear factor-Kappa B
NTA: Nanoparticle tracking analysis
TLR4: Toll-like receptor 4
TEM: Transmission electron microscope
