# Supplementary material for: Human adipose tissue-derived stem cell extracellular vesicles attenuate ocular hypertension-induced retinal ganglion cell damage by inhibiting microglia- TLR4/MAPK/NF-κB proinflammatory cascade signaling
Source: Acta Neuropathol Commun. 2024 Mar 19;12:44. doi: 10.1186/s40478-024-01753-8 (PMC10953184; doi:10.1186/s40478-024-01753-8)
Supplement: Supplementary file 1 — Supplementary Material 1 [file 40478_2024_1753_MOESM1_ESM.pdf]

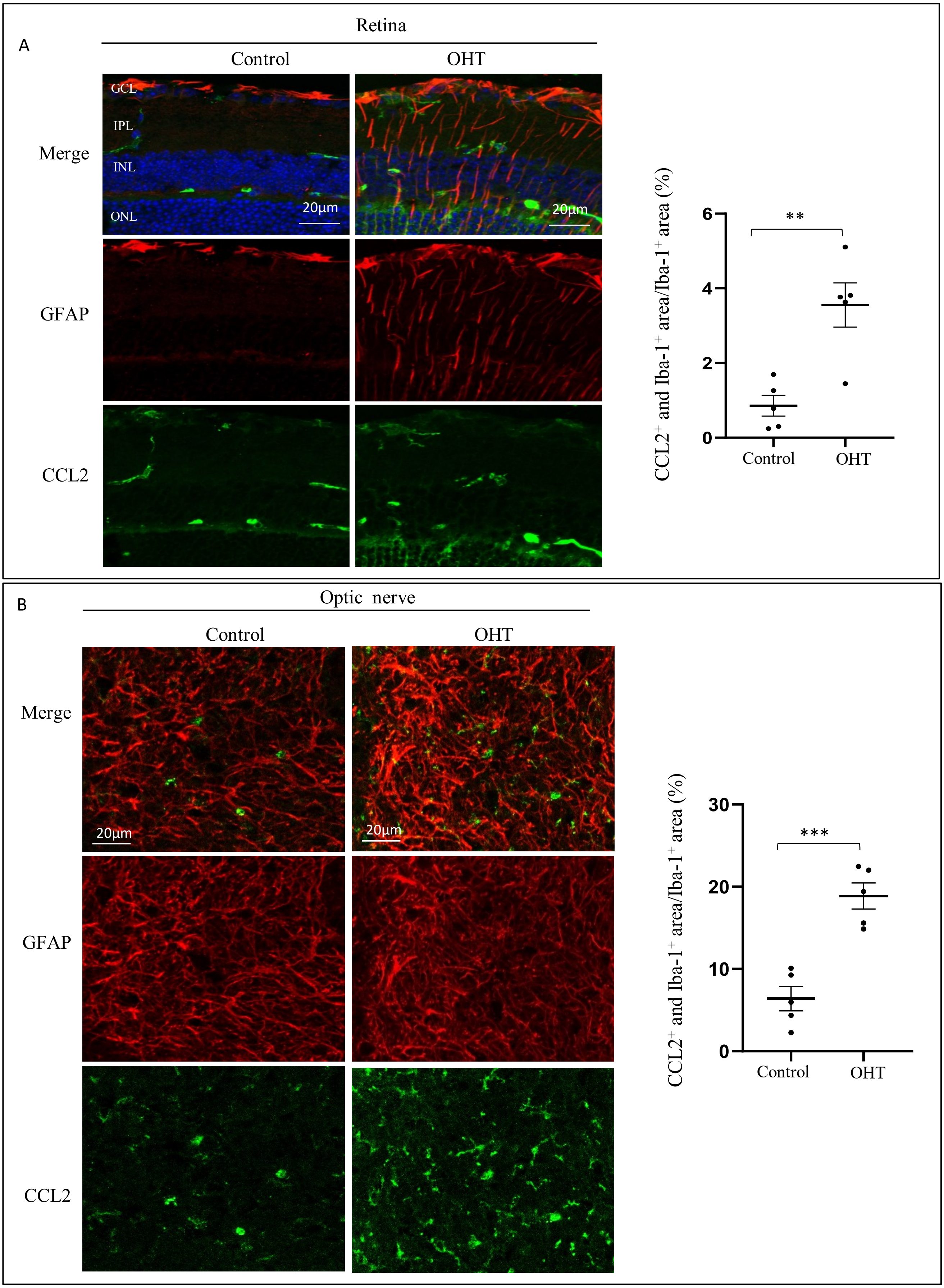

Figure S1. Astrocyte activation and CCL2 expression in OHT mice. (A) Representative double immunofluorescence images of the retina in control and OHT mice at 7 days (n = 5, $^{**}p < 0.01$). (B) Representative double immunofluorescence images of the optic nerve from control and OHT mice at 7 days (n = 5, $^{***}p < 0.001$). GCL, ganglion cell layer; IPL, inner plexiform layer; INL, inner nuclear layer; ONL, outer nuclear layer.
